# Exploring the Impact of Family History, Demographics and Ecological Factors on Urolithiasis Prevalence: Insights from a Nationwide Study

**DOI:** 10.5152/tud.2024.23221

**Published:** 2024-03-01

**Authors:** Abbas Basiri, Amir Hossein Kashi, Mazyar Zahir, Hossein Salehi Omran, Nasrin Borumandnia, Maryam Taheri, Shabnam Golshan, Behzad Narouie, Sakineh Hajebrahimi

**Affiliations:** 1 Urology and Nephrology Research Center, Shahid Labbafinejad Medical Center, Shahid Beheshti University of Medical Sciences, Tehran, Iran; 2 Department of Urology, Zahedan University of Medical Sciences, Zahedan, Iran; 3 Research Center for Evidence-Based Medicine, Iranian EBM Center: A Joanna Briggs Institute Center of Excellence, Tabriz University of Medical Sciences, Tabriz, Iran

**Keywords:** Incidence, Iran, risk factors, urinary calculi, urolithiasis

## Abstract

**Objective:**

This study aimed to evaluate the potential risk factors of lifetime urolithiasis occurrence on a nationwide scale in Iran.

**Methods:**

All data regarding urinary stone events were extracted from the cross-sectional Iran National Stone Survey (INSS) study, and the possible determinants of urolithiasis incidence were evaluated.

**Results:**

Our multivariable logistic regression suggested that while older age at presentation, male sex, and a positive family history of urolithiasis in either of the patient’s parents or siblings were all significantly associated with an increased odds of lifetime urolithiasis occurrence (all *P* < .001), a positive family history in one’s sister (odds ratio; OR = 5.56) or brother (OR = 4.70) were the most significant predictors. Moreover, belonging to Baluch ethnicity (i.e., an ethnical group indigenous to the south eastern regions of Iran) and residing in regions with higher water hardness (i.e., total concentration of dissolved minerals) were also associated with an increased odds of urolithiasis occurrence (*P* < .001 and *P* = .023, respectively). Conversely, living in regions with higher mean humidity decreased the chances of developing a urinary stone event during one’s lifetime (OR = 0.62, *P* < .001).

**Conclusion:**

Our results indicated that a constellation of demographic, ecological, and familial risk factors are associated with an elevated risk of developing urinary stones during one’s lifetime. These findings can assist in implementing novel regional healthcare policies, considering the specific demographic and ecological characteristics. They also support tailoring personalized preventive strategies, particularly for individuals with multiple nonmodifiable risk factors.

**Main Points**

- Older age, male sex, and positive family history, especially among siblings, were identified as significant risk factors of urolithiasis occurrence.
- Baluch ethnicity and residence in regions with higher water hardness were associated with an increased likelihood of lifetime urolithiasis.
- Conversely, living in areas characterized by higher humidity was linked to a decreased risk of developing urinary stones.
- The study sheds light on the intricate interplay of demographic, ecological, and familial elements influencing urolithiasis prevalence.
- With data from over 44 000 participants, this nationwide analysis provides valuable insights into the multifaceted factors contributing to the occurrence of urinary stones.

## Introduction

Urolithiasis is among the most burdensome and challenging urologic conditions globally. According to the latest Global Burden of Diseases study on urolithiasis, the annual incidence of urinary stone diseases exceeded 100 million cases in 2019, and urolithiasis has contributed to approximately 13 000 deaths in the same year.^1^ While recent studies suggest a global decline in urolithiasis incidence, the Middle East and North African region have consistently shown increasing statistics.^1,2^ For instance, in Iran, there has been a 4% rise in the age-standardized urolithiasis incidence rate over the last 3 decades, resulting in an estimated lifetime prevalence of 6.6%.^3,4^

It has been long known that urolithiasis is a multifactorial disease. Previous studies have established the possible role of demographic factors (e.g., age, sex, and race), anthropometric status (e.g., obesity), and positive family history in urolithiasis.^5,6^ Moreover, the potential detrimental influence of dietary factors (e.g., low calcium and high oxalate) and genetic variations (e.g., calcitonin and androgen receptors genes) have been investigated.^7-9^ Nevertheless, evidence regarding the potential impact of ecological factors on urolithiasis risk remains scarce and inconsistent.^10^ Moreover, although previous studies have established a strong association between positive family history and an increased risk of urolithiasis, the differential influence of positive history in either of the first-degree relatives has not been adequately addressed.^5,11,12^

In this study, we combined the data extracted from the Iran National Stone Survey (INSS) study database with regional ecological data (i.e., temperature, rainfall, annual sunshine duration, humidity, and water hardness) obtained from the Iranian national statistics center at the simultaneous time frame. We assessed how these factors impact urinary stone occurrence. Additionally, we investigated the differential influence of a positive history in either of the family members on elevating the risk of urolithiasis.

## Material and Methods

### Study Population and Data Source

All data regarding lifetime prevalence of urolithiasis were extracted from the INSS study database. Iran National Stone Survey was a national epidemiological study on the lifetime prevalence of urinary stones in Iran conducted between October 2020 and November 2022. A detailed description of the INSS study methodology has been previously published.^3^ In summary, a total number of 12 441 families encompassing 44 186 participants were randomly selected from the 31 provinces of Iran and were questioned by telephone interviewers. Phone calls were made proportionate to the ratio of each province’s population to the national population, ensuring that ethnic diversity and respective ethnic ratios of the Iranian population were adequately addressed. Inclusion criteria consisted of being permanent residents of Iran and having a registered telephone line with the Iranian Telecommunications Center. Participants who were unwilling to participate or did not provide the required information about their urolithiasis status were excluded from the study.

In each province, trained local interviewers were in charge of interviewing participants to minimize the possible biases that might have otherwise occurred due to a language barrier. All interviewees were informed that their data would be part of a nationwide study, and they retained the right to withdraw their data within 3 months and prior to the insertion of the data into the finalized data pool. The standard questionnaires used in the study included inquiries about present and past episodes of urinary stone, family history, demographics, and urbanization status. For data quality control, a designated member of the INSS research team rechecked all collected data. If a participant left any question unanswered, the interviewer was directed to make a follow-up call and repeat the specific question. Unanswered questions after the second interview were considered missing data. Furthermore, data for family members who could not be contacted or verified were excluded from the INSS database.

The lifetime prevalence of urolithiasis was defined as any self-reported history of urinary stone passage or positive imaging during the lifespan of the participants.^3^ Data on the mean measurements of ecological variables (i.e., temperature, humidity, rainfall, and annual sunshine duration) were obtained from the Iran National Statistics Center.^13^ Positive history of urolithiasis in either of the household members was also extracted from the INSS database and recorded for further evaluation. The original INSS study was ethically approved by the Iranian National Institute for Medical Research Development (NIMAD) (Approval Code: 989248; IRB Approval ID: IR.NIMAD.REC.1399.113; Date: August 9, 2020).

### Statistical Analysis

All data were expressed as frequency (percentage) for qualitative variables and as mean ± standard deviation (SD) for quantitative variables. Univariable logistic regressions were used to calculate the odds ratio (OR) [95%CI] of potential influential risk factors. To account for the family clusters, a multilevel logistic regression was used to estimate the OR [95% CI] of the explanatory variables for stone occurrence. To assess the potential impact of having a positive family history of stone disease in first-degree relatives, we included familial clusters in the analysis and excluded participants who had missing or unclear family roles in the data record. With respect to ecological variables, we categorized each of them using 5 equal cutoff points based on percentiles of the data distribution. Values below the 16th percentile were assigned to the lowest group, values between the 16th and 84th percentiles to the medium group, and values above the 84th percentile to the highest group. These cutoff points were chosen to capture the variability and skewness of the ecological variables, ensuring each category contained an adequate number of observations for analysis. Moreover, this approach allowed us to compare the effects of extreme values of each of these ecological variables with the central measurements as the reference categories and examine our findings in a biologically more relevant way. A *P* < .05 was considered statistically significant in all analyses. The analysis was performed using Stata software version 14.0 (Stata Corp.; LLC, TX, USA).

## Results

### Demographics and Baseline Characteristics

In total, 44 100 participants were included in our analysis, and 86 participants from the original INSS database were excluded due to their family role being undefined. The lifetime urolithiasis prevalence and 95% CI for each category of the predictors are presented in Table 1. As observed in Table 1, our patient population comprised of 22 043 (50.0%) men and 22 057 (50.0%) women. With regard to age groups, 9771 (22.3%) patients were children and adolescents (i.e., age <18), 27 313 (62.4%) were adults (i.e., age ≥18 and <60), and 6676 (15.3%) were elderly (i.e., age ≥60); according to the definitions proposed by the World Health Organization.^14,15^ A vast majority (73.4%) of our patients lived in urban areas. The most frequently reported ethnicity was Fars (60.0%).

### Demographics and Family History in Urinary Stone Occurrence

The results of our uni- and multivariable analyses are depicted in Table 2. In the univariable analyses, all the variables except for urbanization had a significant *P*-value, meaning that they were associated with urinary stone occurrence. However, in the multivariable analysis, only age, sex, family history, Baluch ethnicity, high humidity, and high water hardness remained significant. As observed, a positive family history in either of the patients’ first-degree relatives was associated with increased odds of developing urolithiasis during lifetime, even after accounting for possible ecologic confounders (all *P* < .001). Similarly, older age and male sex were shown to heighten the odds of developing urinary stone disease in the multivariable model (both *P* < .001). With regard to ethnicity, our primary univariable analyses suggested that 3 of the Iranian ethnicities (i.e., Turk, Lor, and Baluch) were related to increased chances of suffering from urolithiasis. Nevertheless, after entering a multivariable model, the only ethnicity which remained significant as a detrimental factor was the Baluch ethnicity (*P* < .001).

### Ecological Variables and Stone Occurrence

The possible influence of different meteorological factors (i.e., temperature, rainfall, humidity, and annual bright sunshine duration) was also evaluated in this study. We categorized ecological variables into 3 groups based on their values: low, medium (i.e., central measurement), and high. We then used the medium groups as the reference categories and compared the effects of the low and high measurements with the reference categories. For instance, considering temperature, participants were divided into 3 groups based on the mean annual temperature in their residence areas: low (<13.7°C), medium (13.7-18.4°C), and high (>18.4°C). The odds of urolithiasis occurrence were then compared between the low and medium groups, and between the high and medium groups. We did the same for humidity, rainfall, and annual sunshine duration.

Table 2 also shows the results of the univariable and multivariable analyses of the ecological variables and their association with urinary stone occurrence. Our multivariable analysis revealed that after adjusting for other variables, only humidity and water hardness had significant influences on urinary stone occurrence. The odds of urinary stone occurrence were lower for the high humidity group compared to the medium humidity group (OR [95% CI]: 0.62 [0.50, 0.77], *P* < .001). Additionally, the odds of urinary stone occurrence were higher for the high vs. medium water hardness group (OR [95% CI]: 1.21 [1.02, 1.44], *P* = .023). The other ecological variables (i.e., temperature, rainfall, and bright sunshine) did not have any significant effect on urolithiasis in the multivariable analysis. Lastly, urbanization did not affect the chances of lifetime urinary stone disease in either univariable or multivariable analyses.

## Discussion

This study provides a comprehensive picture of the potential demographic, ecological, and familial risk factors associated with increased risk of developing urinary stones on a national level. According to our model, older age, male sex, family history in either of the first-degree relatives, Baluch ethnicity, and residence in areas with higher water hardness were related to an increased odds of developing urolithiasis during a patient’s lifetime. Conversely, residence in more humid areas can potentially decrease the chances of developing urolithiasis during the patient’s lifetime.

The role of demographic risk factors in urolithiasis has been vastly discussed in the literature. Previously, multiple studies have shown that older age is associated with a significantly higher probability of having had at least one episode of urolithiasis in the past.^16,17^ Our analyses revealed a consistent finding, demonstrating an OR [95% CI] of 1.04 [1.04, 1.04] in multivariable analysis. This finding means that with every 1 year increase in age, the odds of urolithiasis increased by 4%. This is consistent with the finding that urolithiasis prevalence is higher in the elderly population compared to younger people. Similarly, with regard to the role of patient’s sex on the lifetime prevalence of urolithiasis, our results were in concordance with the literature,^16,18^ demonstrating an approximately 50% more chance of suffering from urolithiasis during lifetime among men. This finding also suggests an increase in the male-to-female ratio of lifetime urolithiasis in Iran compared to the previous national study which reported a 1.15 ratio.^19^

According to our multivariable analysis, a positive family history of urolithiasis in the patient’s brother, sister, father, and mother increased the odds of lifetime urinary stone occurrence 4.7-, 5.6-, 2.4-, and 2.3-fold, respectively. Consequently, a positive history in the patient’s siblings appears to have a more deleterious effect on stone occurrence compared to a positive history in the patient’s parents. In line with this finding, a previous study from Sweden found that the standardized incidence ratio of urolithiasis in patients with a positive family history in 2 of their siblings was noticeably higher than those with a positive family history in both parents (24.91 vs. 3.94, respectively).^20^ However, in a study by Edvardsson et al, contradictory results were reported, indicating a more prominent risk with a positive parental history compared to a positive sibling history.^21^ Nevertheless, it is noteworthy that the latter study was conducted in Iceland, a small country with a relatively low population and a high level of consanguinity; factors which may hinder the generalizability of their findings.^22^

Regarding the possible contribution of ethnicity to an increased risk of urolithiasis, Baluch ethnicity was the only factor associated with an approximately 2-fold increase in urolithiasis occurrence. The higher prevalence of urolithiasis in the southeastern region of Iran was first reported by Safarinejad et al in 2007.^19^ Nevertheless, the role of ethnicity was not specifically investigated in that study, and the main point of focus was the geographical divisions. The INSS study was the first nationwide study to underscore the higher risk of urolithiasis in Baluch ethnicity.^3^ However, the INSS study suggested that other than Baluch ethnicity, Kurd and Lor ethnicities could also increase the chances of urinary stone occurrence. This discrepancy may be partly explained by the wider range of variables (e.g., family history and ecological factors) incorporated in our statistical model compared to the INSS study. The higher prevalence of urolithiasis observed among Baluch people may be attributed to risk factors unaccounted for in this study, such as the lower socioeconomic development in Sistan and Baluchestan province and also the established genetic distinction between Baluch ethnicity and the other Iranian ethnicities.^23,24^

We also evaluated the possible contribution of ecological risk factors to lifetime urolithiasis occurrence. Initially, our univariable analyses suggested that the extremes of different ecological factors (i.e., temperature, rainfall, bright sunshine, and humidity) are associated with increased odds of suffering from a urinary stone event. Nevertheless, after incorporating all these variables in a multivariable predictive model, the only variable which demonstrated statistical significance was annual mean humidity. Our data suggested that a higher humidity level is associated with a 38% decrease in the odds of suffering from urolithiasis. This finding was predictable since atmospheric humidity levels can affect perspiration, which is an established risk factor of urolithiasis.^25^

Lastly, we also evaluated the potential detrimental effect of water hardness on urolithiasis incidence. Our findings indicated that living in areas where total water hardness is very high (>570 ppm) is associated with an approximately 20% increase in the odds of a lifetime urolithiasis event. This finding was in line with a former cross-sectional study in southeastern Iran, which demonstrated that drinking unpurified hard water is associated with a 20% increase in the lifetime urolithiasis occurrence compared to drinking purified soft water.^18^ There has been an ongoing debate regarding the potential role of water hardness on kidney stone disease with different studies showing contradictory findings. The controversy regarding the positive or negative role of water hardness on urolithiasis dates back to almost half a century ago when 2 large studies, one conducted in the United States and the other in the United Kingdom, reported exactly opposite findings.^26,27^ Nevertheless, newer studies based on much more robust statistical methods mostly refute the role of water hardness on kidney stone disease.^28,29^ These paradoxical findings urge further studies to better address the possible role of water hardness on urolithiasis occurrence.

Our study had some limitations. First, the cross-sectional study design hinders the inference of any causal relationships. Additionally, the data are derived from patient-reported urolithiasis events which may introduce recall bias and possible under- or over-reporting of kidney stone events. Moreover, we combined these patient-derived data with large-scale national ecological factors, which may have affected our results. Lastly, it is worthy of mentioning that there is inevitable collinearity between different ecological factors (e.g., rainfall and humidity). Nevertheless, considering the very large sample size in our study and comprehensive data collection for the associated risk factors of interest, our findings are worthy of consideration.

In conclusion, in this study, we demonstrated that a constellation of demographic (i.e., age, sex, and ethnical background), ecological (i.e., water hardness and mean humidity), and familial (i.e., family history in either of the parents or siblings) risk factors are associated with an elevated risk of developing urinary stones during one’s lifetime.

## Figures and Tables

**Table 1.** The Lifetime Prevalence of Urinary Stone Categorized by Different Baseline Characteristics

Variables		Total Participants	Urolithiasis Frequency	95.% CI
Age (years)	<18	9771	85 (0.9%)	0.7%-1.1%
	≥18, <60	27 313	1944 (7.1%)	6.8%-7.4%
	≥60	6676	830 (12.4%)	11.7%-13.2%
Sex	Female	22 043	1167 (5.3%)	5.0%-5.6%
	Male	22 057	1728 (7.8%)	7.5%-8.2%
Positive family history	Father	2934	299 (10.2%)	9.1%-11.3%
	Mother	1912	221 (11.6%)	10.2%-13.0%
	Sister	675	257 (38.1%)	34.5%-41.8%
	Brother	1213	379 (31.2%)	28.7%-33.9%
Ethnicity	Fars	26 415	1595 (6.0%)	5.8%-6.3%
	Turk	8502	589 (6.9%)	6.4%-7.5%
	Lor	3419	250 (7.3%)	6.5%-8.2%
	Kurd	3354	195 (5.8%)	5.1%-6.6%
	Arab	1092	59 (5.4%)	4.2%-6.9%
	Baluch	1068	192 (18.0%)	15.8%-20.4%
	Others	194	12 (6.2%)	3.4%-10.2%
Urbanization	Rural	11 719	797 (6.8%)	6.4%-7.3%
	Urban	32 381	2098 (6.5%)	6.2%-6.8%

**Table 2.** Possible Contributors to Lifetime Occurrence of Urolithiasis

		Univariable Analysis		Multivariable Analysis	
		Odds Ratio (95% CI)	*P*	Odds Ratio (95% CI)	*P*
Age at the time of the study		1.03 (1.03, 1.03)	<.001	1.04 (1.04, 1.04)	<.001
Sex (ref: female)	Male	1.52 (1.40, 1.64)	<.001	1.48 (1.36, 1.61)	<.001
Positive family history (ref: negative)	Father	1.68 (1.48, 1.91)	<.001	2.40 (2.07, 2.79)	<.001
	Mother	1.93 (1.67, 2.23)	<.001	2.33 (1.96, 2.77)	<.001
	Sister	9.50 (8.09, 11.15)	<.001	5.56 (4.61, 6.71)	<.001
	Brother	7.29 (6.41, 8.28)	<.001	4.70 (4.60, 5.44)	<.001
Ethnicity (ref: Fars)	Turk	1.15 (1.05, 1.27)	.003	1.09 (0.94, 1.25)	.219
	Lor	1.22 (1.06, 1.41)	.004	1.13 (0.94, 1.36)	.180
	Kurd	0.96 (0.82, 1.12)	.607	0.89 (0.72, 1.10)	.292
	Arab	0.88 (0.68, 1.16)	.387	0.88 (0.63, 1.22)	.465
	Baluch	3.41 (2.89, 4.01)	<.001	2.07 (1.58, 2.70)	<.001
	Others	1.02 (0.57, 1.84)	.932	1.08 (0.57, 2.07)	.794
Urbanization (ref: urban)	Rural	1.05 (0.96, 1.14)	.228	1.0 (0.91, 1.10)	.916
Temperature (°C) (ref: >13.7 and <18.4)	<13.7	1.14 (1.03, 1.26)	.007	1.10 (0.92, 1.31)	.259
	>18.4	1.37 (1.24, 1.52)	<.001	1.02 (0.86, 1.20)	.808
Rainfall (mm) (ref: >98.3 and <373.5)	<98.3	1.52 (1.38, 1.66)	<.001	1.34 (0.98, 1.84)	.061
	>373.5	0.96 (0.86, 1.07)	.507	1.14 (0.94, 1.39)	.173
Bright sunshine (hours) (ref: >2825.4 and <3354.6)	<2825.4	1.02 (0.91, 1.13)	.636	1.16 (0.93, 1.54)	.073
	>3354.6	1.62 (1.46, 1.80)	<.001	1.08 (0.83, 1.40)	.559
Humidity (%) (ref: >34.5 and <58.5)	<34.5	1.51 (1.38, 1.65)	<.001	0.92 (0.66, 1.28)	.641
	>58.5	0.82 (0.73, 0.92)	.001	0.62 (0.50, 0.77)	<.001
Water hardness (ppm) (ref: >378 and <570)	<378	1.05 (0.95, 1.15)	.272	0.96 (0.84, 1.09)	.575
	>570	1.57 (1.43, 1.73)	<.001	1.21 (1.02, 1.44)	.023
